# The Molecular Characterization and Antioxidant Defense of a Novel Nrf2 from the Pacific Abalone *Haliotis discus hannai* Ino

**DOI:** 10.3390/ijms252212429

**Published:** 2024-11-19

**Authors:** Kun Qiao, Qiongmei Huang, Bei Chen, Min Xu, Hua Hao, Yongchang Su, Shuji Liu, Nan Pan, Zhiyu Liu

**Affiliations:** 1 Key Laboratory of Cultivation and High-Value Utilization of Marine Organisms in Fujian Province, Fisheries Research Institute of Fujian, Xiamen 361013, China; qiaokun@xmu.edu.cn (K.Q.); 13950001893@163.com (B.C.); xumin@jmu.edu.cn (M.X.); suyongchang@126.com (Y.S.); cute506636@163.com (S.L.); npan01@qub.ac.uk (N.P.); 2 College of Food Science, Fujian Agriculture and Forestry University, Fuzhou 350002, China; 1220919043@fafu.edu.cn; 3 College of Ocean and Earth Sciences, Xiamen University, Xiamen 361013, China; hhao@xmu.edu.cn

**Keywords:** *Haliotis discus hannai* Ino, Nrf2, antioxidant, oxidative stress

## Abstract

The Nrf2/ARE pathway is considered the most important endogenous antioxidant signaling pathway in mammals, playing a crucial role in defending against external damage. This study investigated the functional characteristics of Nrf2 in the abalone, *Haliotis discus hannai*. The full-length cDNA sequence of the *HdhNrf2* gene was cloned using rapid amplification of cDNA ends (RACE) technology and consists of 4568 base pairs encoding a protein of 694 amino acids. The predicted theoretical molecular weight was 77 kDa, with an isoelectric point of 4.72. Multiple sequence alignment analysis revealed the relative conservation of the HdhNrf2 amino acid sequence in *H. discus hannai*. The tissue expression pattern of the *HdhNrf2* gene was analyzed using real-time fluorescence quantitative PCR, which showed the highest expression in the gills, followed by hemocytes, with the lowest levels in the foot and mantle. The inducible expression of HdhNrf2 and antioxidant genes in abalone under H_2_O_2_ stress was investigated at various time points. Furthermore, an expression vector, pET-28a(+)-rHdhNrf2, was constructed, and the recombinant protein rHdhNrf2 was obtained through induced expression and purification. These findings indicated that HdhNrf2 plays a crucial role in the defense of abalones against oxidative stress.

## 1. Introduction

Free radicals are atoms, molecules, ions, or atomic groups with unpaired electrons, and are indispensable active substances in organisms. Among the free radicals, reactive oxygen species (ROS) and their related intermediates cause the most damage to organisms and are the most well-studied [1]. ROS are produced by cellular metabolism and include free radicals, such as the superoxide anion (O_2_^−^) and hydroxyl radical (OH), singlet oxygen (1O_2_), and hydrogen peroxide (H_2_O_2_) [2]. Under normal physiological and metabolic conditions, ROS exist at an equilibrium in organisms. However, when invaded by pathogens, the host is stimulated by a series of inflammatory reactions that produce ROS during phagocytosis, a process known as the respiratory burst [3]. Researchers believe that in crustacean hemocytes, lysosomes and multiple ROS produced during phagocytosis constitute the main mechanism of bactericidal action and that ROS can enhance host immunity [4]. However, many exogenous stimuli, such as ultraviolet radiation, environmental toxins, high temperatures, and hypoxia, can stimulate cells to produce large amounts of ROS, resulting in cellular oxidative damage. Excessive ROS can lead to the degradation of unsaturated fatty acids in biofilms, the denaturation of proteins, changes in membrane structure, and the inactivation of enzymes, eventually causing DNA strands to break [5,6].

Nuclear factor-erythroid 2 related factor 2/antioxidant response element (Nrf2/ARE) is considered the most important endogenous antioxidant signaling pathway in mammals, as it is resistant to the oxidative stress caused by endogenous, exogenous, and chemical stimuli [7,8]. Nrf2 is an important transcription factor that regulates cellular antioxidant responses and belongs to the Cap’-N’-Collar (CNC) transcription factor family [7]. Under normal physiological conditions, Nrf2 binds to the cytosolic chaperone protein, Kelch-like ECH-associated protein1 (Keapl), in a relatively inhibitory state and is anchored in the cytoplasm. When subjected to oxidants or electrophiles, Nrf2 is uncoupled from Keap1 and undergoes a series of protein kinase phosphorylations, which causes Nrf2 to be transferred into the nucleus, where it binds to a Mar protein and forms a heterodimer. This heterodimer, which recognizes and binds to ARE, initiates the transcription of antioxidant genes and those encoding several phase II detoxifying enzymes, thereby exerting a strong antioxidant effect [9]. The genes encoding phase II detoxification enzymes and antioxidant proteins of the Nrf2/ARE pathway mainly include heme oxygenase-1 (HO-1), glutathione-S-transferase (GST), catalase (CAT), superoxide dismutase (SOD), and NADP(H) 3 oxidoreductase (quinone oxidoreductase 1, NQO1) [10]. When cells return to redox homeostasis, the Nrf2 signaling pathway is shut down, Keap1 enters the nucleus, and Nrf2 is removed from the ARE sequence, forming a complex that is transferred out of the nucleus where it returns to its normal physiological state [11].

Among marine organisms, the Nrf2/ARE signaling pathway has mainly been studied in fish. Kobayashi et al. [12] first described the Nrf2/ARE signaling pathway in zebrafish. Zebrafish and mammalian Nfr2 have many similarities, proving that the pathway is conserved in vertebrates [12]. Two years later, the team discovered three other important components of the Nrf2/ARE signaling pathway: Maf G, Maf K, and Maf T [13]. Two homologous genes (Nrf2a and Nrf2b) have been found in zebrafish, most of which are conserved in both zebrafish Nrf2 proteins, whereas Nrf2b lacks the Neh4 transactivation domain found in Nrf2a and mammalian Nrf proteins. The primary role of Nrf2a is as a constitutive activator of transcription, whereas Nrf2b is primarily involved in the negative regulation of gene expression in embryos [14,15]. Giuliani and Regoli [16] cloned the *Nrf2* and *Keap1* genes of *Anguilla anguilla* and studied the transcriptional regulation of antioxidant genes of the *Keap1-Nrf2* signaling pathway in H_2_O_2_-induced peroxidative damage. Studies on the Nrf2/ARE signaling pathway have mainly focused on fish peroxidative damage caused by environmental factors. Jiang et al. [17] reported the toxic effects of copper exposure on the Nrf2/ARE signaling pathway and caspase-3-mediated regulation of the antioxidant system. The regulation of the Nrf2-Keap1 signaling pathway in peroxidative damage caused by zinc exposure was further explored by Zheng et al. in large yellow croakers (*Pseudosciaena crocea*) [18]. However, limited information is available on the antioxidant signaling pathways in marine invertebrates. Nrf2 in marine invertebrates was not reported until recently, and with the rapid development of sequencing technology, the key genes regulating the Nrf2/ARE pathway in marine invertebrates have been described. Danielli et al. [19,20] first described the *Nrf2* and *Keap1* genes in *Crassostrea gigas*, and later Wang et al. [21] confirmed the existence of the Nrf2/ARE signaling pathway in *Ruditapes philippinarum*.

The Pacific abalone (*Haliotis discus hannai* Ino.) contributes substantially to China’s aquaculture economy. The abalone industry has become one of the most important industries for marine fisheries in Fujian Province, where the production of *H. discus hannai* is extensive. In recent years, cultured abalones have shown low resistance to disease owing to the quality of water used in abalone farming areas and the degeneration of cultured species, which may result in enormous economic losses, especially during disease outbreaks. The occurrence of disease outbreaks in cultured abalones is closely related to changes in environmental factors [22]. Addressing the knowledge gap in understanding Nrf2 in marine invertebrates, this study posits the hypothesis that the abalone possesses an Nrf2/ARE signaling pathway, which may play a crucial role in its antioxidant defense system, particularly in response to oxidative stress induced by environmental changes. To test this hypothesis, we successfully cloned the HdhNrf2 gene and, for the first time, analyzed its differential expression under H_2_O_2_-induced oxidative stress, aiming to uncover the functional characteristics of Nrf2 in the abalone’s antioxidant response. To our knowledge, this is the first characterization of Nrf2 in Gastropoda, and this work will facilitate our understanding of the functional role of the Nrf2/ARE signaling pathway in invertebrates.

## 2. Results

### 2.1. Molecular Characteristics of the HdhNrf2 Gene in H. discus hannai

Open reading frames (ORFs) of *HdhNrfr2* were obtained from the transcriptome database of *H. discus hannai*, which was previously constructed in our laboratory. The 5′ and 3′ untranslated sequences obtained by rapid amplification of cDNA ends (RACE) were further spliced to obtain a full-length cDNA of 4568 bp (GenBank accession no. MK848864), with a 65 bp 5′ untranslated region and a 2418 bp 3′ untranslated region with a typical poly(A) tail (Supplementary Figure S1a). The ORFs of *HdhNrf2* cDNA were predicted using DNASIS MAX V3.0 to encode a polypeptide chain consisting of 694 amino acids. The theoretical molecular weight was predicted to be 77 kDa with an isoelectric point of 4.72 and was computed using the ExPASy ProtParam tool (http://web.expasy.org/protparam, accessed on 17 November 2024). Conserved domains were analyzed using an NCBI CD search. In the HdhNrf2 protein of *H. discus hannai*, the basic leucine zipper (bZIP) domain of CNC transcription factor appears at amino acids 570–637, which is conserved when compared with other species (Supplementary Figure S1b).

### 2.2. Multi-Sequence Alignment Comparison and Evolution Analysis of HdhNrf2

**Figure 1:**
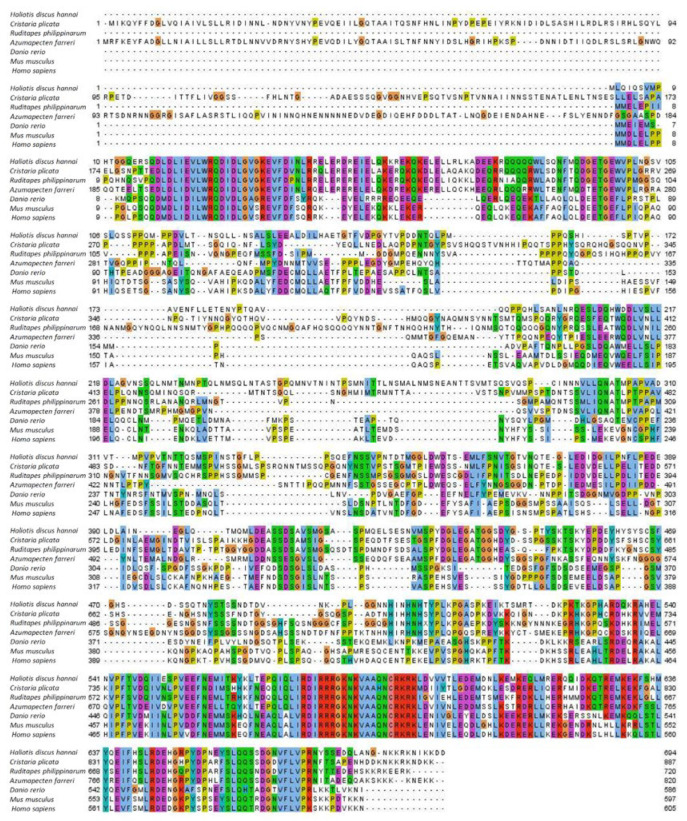
Multiple sequence alignment of *Haliotis discus hannai* (MK848864) and *Cristaria plicata* (AZM32563.1), *Ruditapes philippinarum* (AWV55267.1), *Azumapecten farreri* (AWV55266.1), *Danio rerio* (BAC10573.1), *Mus musculus* (NP_035032.1) and *Homo sapiens* (NP 006155.2) Nrf2 proteins. Identical amino acids are shaded in black and similar amino acids are shaded in gray. The numbers on the left and right show the aa position within the corresponding species.

**Figure 2:**
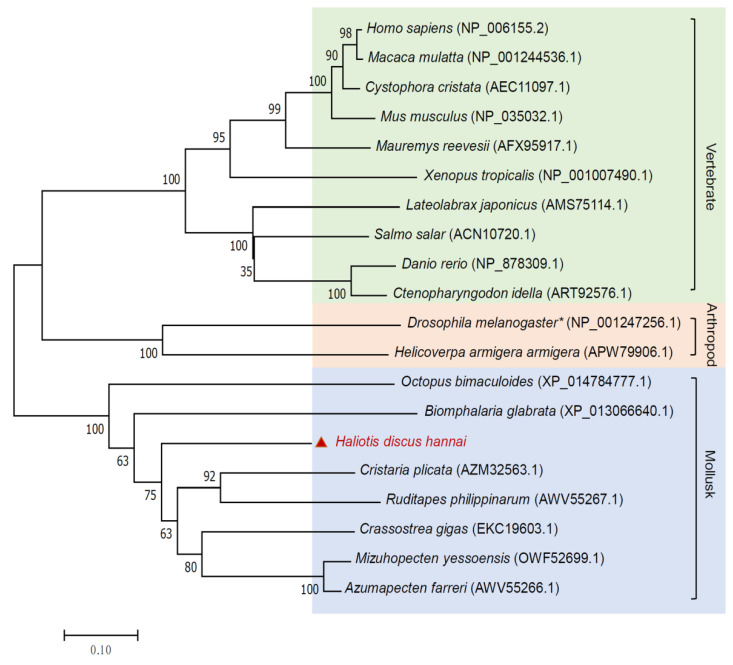
Neighbor-joining phylogenetic tree based on multiple alignments (ClustalW) of Nrf2 amino acid sequences from various species. One thousand bootstrap trials were run using the neighbor-joining algorithm using the MEGA7.0 software. The numbers shown at the branches denoted the bootstrap majority consensus values of 1000 replicates. The number after the Latin name indicates the GenBank accession number. The triangle symbol represents the abalone *Haliotis discus hannai*.

The *Nrf2* gene sequences of other species were obtained from GenBank. A multi-sequence alignment comparing the predicted HdhNrf2 amino acid sequence with the known *Nrf2* gene of other species is shown in Figure 1. Alignment analyses revealed conserved domains in different species, suggesting a conserved binding region (RRRGKNKVAQHNCRKR) in the bZIP_CNC domain of abalone. According to a comparison using NCBI BLASTP (https://blast.ncbi.nlm.nih.gov/Blast.cgi, accessed on 17 November 2024), the HdhNrf2 amino acid sequence was relatively conserved in *H. discus hannai*, with 25–95% amino acid coverage and 40–52.14% identity (Table 1. The phylogenetic tree created using Molecular Evolutionary Genetics Analysis (MEGA) 7.0 software contained 20 species with Nrf2 proteins, including vertebrates and invertebrates (Figure 2). The *Nrf2* genes of each species were clustered into different clades. A phylogenetic analysis revealed three major clades: vertebrates, mollusks, and a third one for arthropods. *H. discus hannai* Nrf2 was closer to that of Bivalvia, including *Cristaria plicata*, *Ruditapes philippinarum*, *Crassostrea gigas*, *Mizuhopecten yessoensis*, and *Azumapecten farreri.*

### 2.3. Distribution of HdhNrf2 in Tissues of H. discus hannai

**Figure 3:**
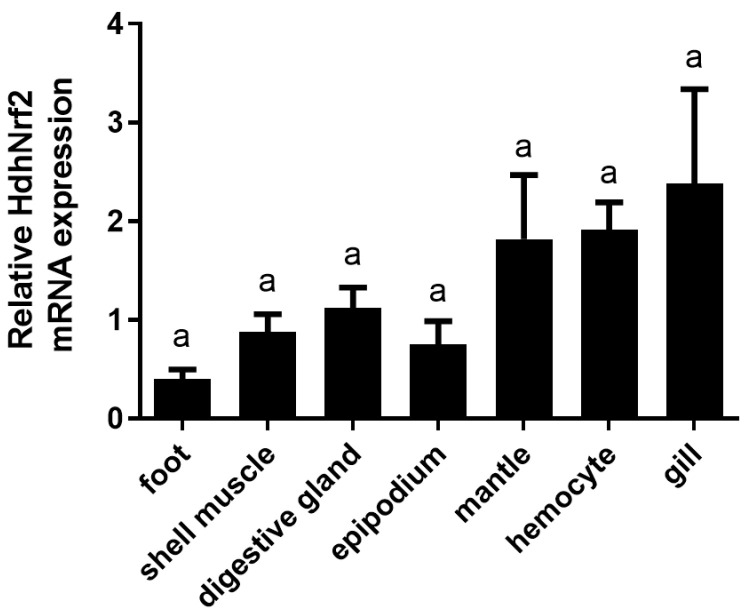
The distribution of *HdhNrf2* mRNA transcripts and protein in different tissues of normal *H. discus hannai. RPL3* was used as a house-keeping gene. Error bars represent the SD. Mean values and SD are shown from four biological replicates. The bars show the standard errors of mean values. Different letters indicate significant differences among control and treatments (*p* < 0.05).

qPCR was performed to investigate the transcript distribution of *HdHNrf2* in various tissues. *HdHNrf2* mRNA had a broad tissue distribution, including the hemocytes, gills, mantle, digestive glands, and shell muscles. The expression levels are the highest in tissues that play a key role in antioxidant defense (such as gills and hemocytes). The results indicate that the Nrf2 gene exhibits constitutive expression among various tissues in abalone, with no significant differences in gene expression levels among the tissues (Figure 3).

### 2.4. Quantitative Analysis of the HdhNrf2 and Antioxidant Genes Expression After H_2_O_2_ Challenge

**Figure 4:**
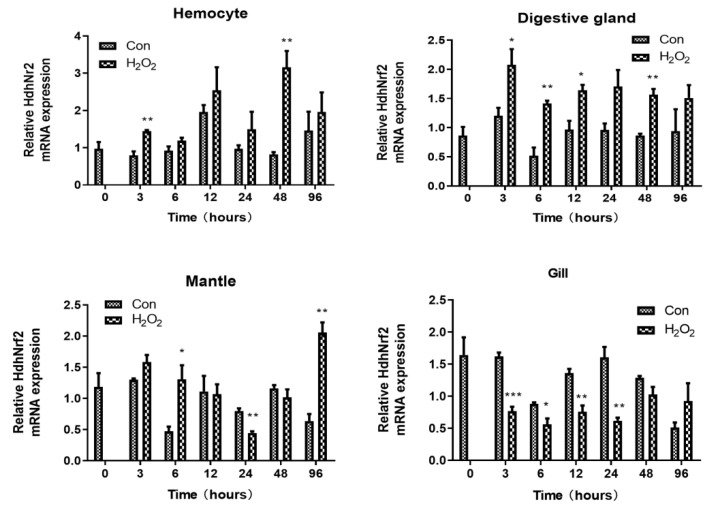
The mRNA expression of *HdhNrf2* in hemocytes, gill, digestive gland, and mantle over time after H_2_O_2_ challenge. *RPL7* was used as a house-keeping gene. Error bars represent the SD. Mean values and SD are shown from three biological replicates. Independent t tests demonstrated significant difference between H_2_O_2_ and control groups with df = 4. The level of significance is indicated with asterisks (* *p* < 0.05; ** *p* < 0.01; *** *p* < 0.001).

**Figure 5:**
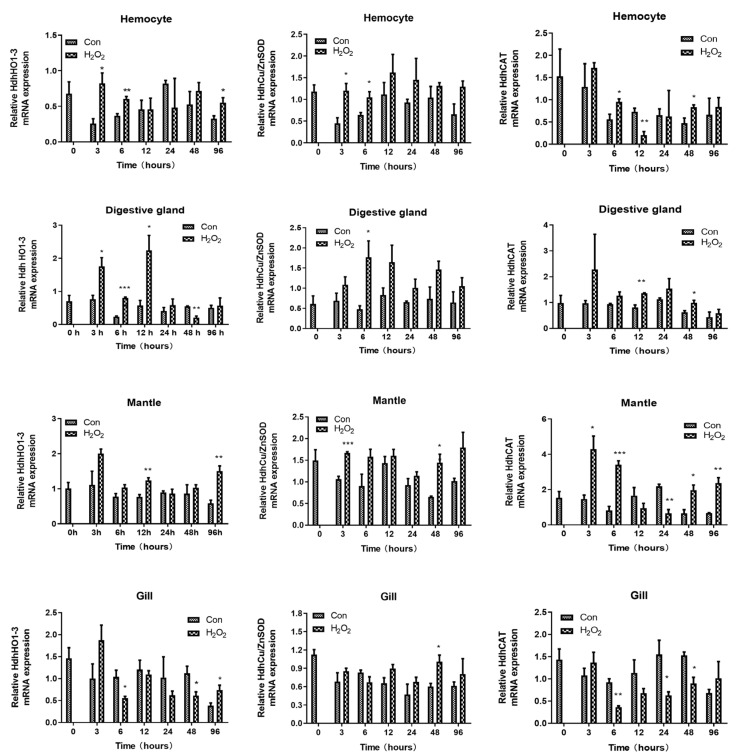
Transcript levels of antioxidant enzymes after H_2_O_2_ challenge in hemocyte, digestive gland, mantle and gill. *RPL7* was used as a house-keeping gene. Error bars represent the SD. Mean values and SD are shown from three biological replicates. Independent t tests demonstrated significant differences between H_2_O_2_ and control groups with df = 4. The level of significance is indicated with asterisks (* *p* < 0.05; ** *p* < 0.01; *** *p* < 0.001).

qPCR was employed to determine the expression levels of *HdhNrf2* and other antioxidant genes in response to H_2_O_2_ challenge. As shown in Figure 4, the transcripts of *HdhNrf2* in hemocytes were significantly upregulated at 3 h (F-statistic = 2.035; *p* = 0.004) and 48 h post challenge (F-statistic = 2.894; *p* = 0.006). *HdhNrf2* expression levels in the digestive gland were significantly upregulated at 3 h (F-statistic = 3.055; *p* = 0.045), 6 h (F-statistic = 4.974; *p* = 0.004), 12 h (F-statistic = 1.606; *p* = 0.019), and 48 h (F-statistic = 3.370; *p* = 0.003) post challenge (Figure 5). In the mantle, *HdhNrf2* was significantly upregulated at 6 h (F-statistic = 3.697; *p* = 0.024) and 96 h (F-statistic = 0.222; *p* = 0.002) and downregulated at 24 h (F-statistic = 1.252; *p* = 0.002). In the gills, *HdhNrf2* was downregulated at most time points, with significant differences at 3 h (F-statistic = 0.000; *p* = 0.001), 6 h (F-statistic = 6.826; *p* = 0.028), 12 h (F-statistic = 0.337; *p* = 0.007), and 24 h (F-statistic = 6.415; *p* = 0.004) post challenge. These results suggest that a tissue- and time-dependent expression pattern of *HdhNrf2* occurred in abalones in response to H_2_O_2_ challenge.

**Figure 6:**
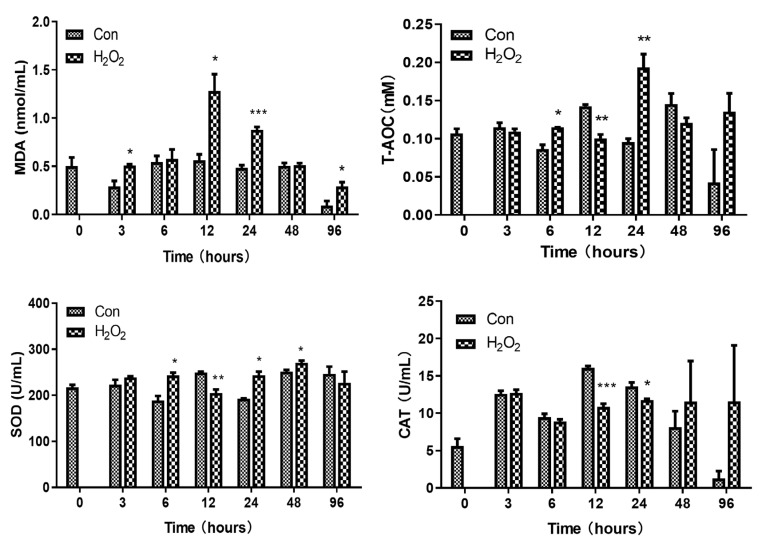
Antioxidant enzyme activity after H_2_O_2_ challenge in serum. Error bars represent the SD. Mean values and SD are shown from three biological replicates. Independent t tests demonstrated significant differences between H_2_O_2_ and control groups with df = 4. The level of significance is indicated with asterisks (* *p* < 0.05; ** *p* < 0.01; *** *p* < 0.001).

The mRNA expression levels of *HdhCu/ZnSOD*, *HdhCAT*, and *HdhHO1* after H_2_O_2_ treatment are shown in Figure 6. In hemocytes, *HhdHO1* expression was significantly upregulated at 3 h (F-statistic = 3.154; *p* = 0.026), 6 h (F-statistic = 0.375; *p* = 0.007), and 96 h (F-statistic = 1.610; *p* = 0.045). A similar trend was observed for *HdhCu/ZnSOD* expression at 3 h (F-statistic = 0.297; *p* = 0.023) and 6 h (F-statistic = 3.724; *p* = 0.044). Notably, the transcript levels of *HdhCAT* were markedly downregulated at 12 h (F-statistic = 0.084; *p* = 0.009) and upregulated at 6 h (F-statistic = 0.710; *p* = 0.04) and 48 h (F-statistic = 1.436; *p* = 0.046).

In the digestive gland, *HhdHO1* expression was significantly upregulated at 3 h (F-statistic = 3.837; *p* = 0.026), 6 h (F-statistic = 0.06; *p* = 0.000), and 12 h (F-statistic = 5.161; *p* = 0.026) but downregulated at 48 h (F-statistic = 6.635; *p* = 0.003). *HdhCu/ZnSOD* expression was upregulated at 6 h (F-statistic = 8.976; *p* = 0.037). *HdhCAT* was markedly upregulated at 12 h (F-statistic = 5.190; *p* = 0.005) and 48 h (F-statistic = 1.426; *p* = 0.037).

*HhdHO1* mRNA expression in the mantle was significantly upregulated in the control group at 12 h (F-statistic = 0.203; *p* = 0.009) and 96 h (F-statistic = 0.486; *p* = 0.006). The transcript levels of *HdhCu/ZnSOD* were markedly upregulated at 3 h (F-statistic = 2.050; *p* = 0.001) and 48 h (F-statistic = 11.059; *p* = 0.015). *HdhCAT* was downregulated at 24 h (F-statistic = 2.070; *p* = 0.003) and significantly upregulated at 3 (F-statistic = 6.790; *p* = 0.023), 6 h (F-statistic = 0.034; *p* = 0.001), 48 h (F-statistic = 0.608; *p* = 0.02), and 96 h (F-statistic = 6.428; *p* = 0.005).

In the gill, *HdhHO1* was downregulated at 6 h (F-statistic = 3.669; *p* = 0.036) and 48 h (F-statistic = 2.426; *p* = 0.049), and upregulated at 96 h (F-statistic = 1.902; *p* = 0.046). *HdhCu/ZnSOD* was significantly upregulated at 48 h (F-statistic = 3.652; *p* = 0.028). The *HdhCAT* mRNA expression pattern was similar to that of *HdhHO1* with the challenge of H_2_O_2_, which showed a significantly downregulated expression level from the control group at 6 h (F-statistic = 6.394; *p* = 0.003), 24 h (F-statistic = 2.967; *p* = 0.049), and 48 h (F-statistic = 1.790; *p* = 0.015). These results indicate that the Nrf2/ARE signaling pathway was induced by H_2_O_2_.

The total antioxidant capacity (T-AOC), CAT, SOD, and malondialdehyde (MDA) activities in the serum of H_2_O_2_-stressed abalones were measured at 3, 6, 12, 24, 48, and 96 h. The results showed that MDA levels were significantly higher at 3 h (F-statistic = 4.790; *p* = 0.02), 12 h (F-statistic = 5.454; *p* = 0.017), 24 h (F-statistic = 0.003; *p* = 0.001), and 96 h (F-statistic = 0.076; *p* = 0.039) of H_2_O_2_ stress, indicating that substantial oxidative damage occurred in the H_2_O_2_-stressed wrinkled abalone. The T-AOCs of H_2_O_2_-stressed abalones (Figure 6) were significantly increased in the H_2_O_2_-stressed group at 6 h (F-statistic = 14.687; *p* = 0.37) and 24 h (F-statistic = 2.836; *p* = 0.006) compared with that in the corresponding saline control group, but decreased significantly at 12 h (F-statistic = 3.184; *p* = 0.002) post injection. SOD activity was significantly increased at 6 h (F-statistic = 0.662; *p* = 0.01), 24 h (F-statistic = 9.051; *p* = 0.026), and 48 h (F-statistic = 0.391; *p* = 0.039) compared with that in the control group, and was significantly decreased at 12 h (F-statistic = 7.167; *p* = 0.005) in the serum of H_2_O_2_-stressed abalone. CAT activity in the serum of H_2_O_2_-stressed abalones at 12 h (F-statistic = 0.735; *p* = 0.000) and 24 h (F-statistic = 6.794; *p* = 0.030) was significantly lower than that of the control group.

### 2.5. Expression and Purification of pET28a-Nrf2 Recombinant Protein

A sufficient amount of the target product was amplified by multiple rounds of PCR, and the expression vector pET28a was double-digested with *Nde I* and *Xho I* (Figure S2) and transformed by ligation with the purified target DNA fragment to screen positive clones. The fusion protein expressed by this expression vector has a 6× His tag attached to the amino terminus of the target protein, so that the fusion protein can be purified using a Ni+ affinity chromatography column.

**Figure 7:**
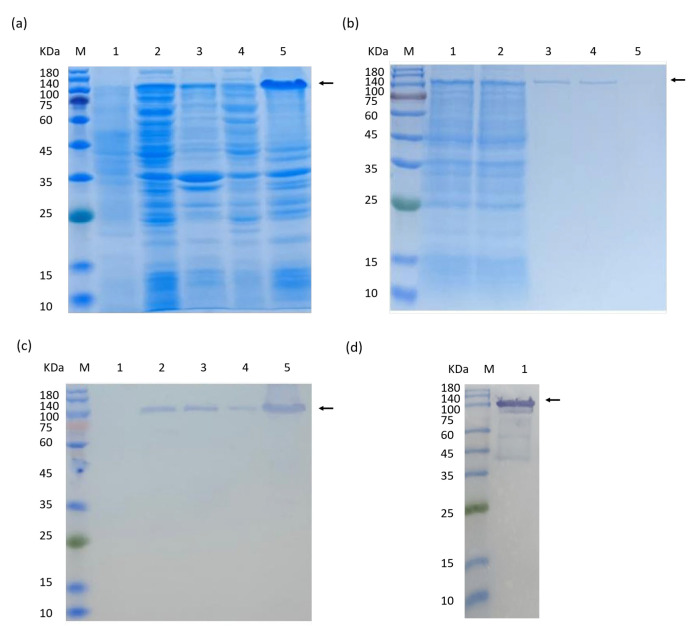
The recombinant expression and purification of rHdhNrf2. (**a**) SDS-PAGE analysis of the rHdhNrf2 protein expression optimization. lane M: Protein Marker (Cat. No.: C610013); lane 1, recombinant *E. coli* BL21 (DE3) strain without IPTG; lane 2, lysate soluble fraction from *E. coli* cultivation at 20 °C; lane 3, lysate pellets after sonication from *E. coli* cultivation at 20 °C; lane 4, lysate soluble fraction from *E. coli* cultivation at 37 °C; lane 5, lysate pellets after sonication from *E. coli* cultivation at 37 °C. (**b**) SDS-PAGE analysis of the purified rHdhNrf2 protein. lane M: Protein Marker (Cat. No.:C610013); lane 1, total protein content before purification; lane 2, outflow component; lane 3, 20 mmol/L imidazole elution component; lane 4; 50 mmol/L imidazole eluting component; lane 5, 500 mmol/L imidazole eluting component. (**c**) Western blot analysis of rHdhNrf2 protein expression. lane M: Protein Marker (Cat. No.: C610013); lane 1, recombinant *E. coli* BL21 (DE3) strain without IPTG; lane 2, lysate soluble fraction from E. coli cultivation at 20 °C; lane 3, lysate pellets after sonication from E. coli cultivation at 20 °C; lane 4, lysate soluble fraction from E. coli cultivation at 37 °C; lane 5, lysate pellets after sonication from *E. coli* cultivation at 37 °C. (**d**) Western blot analysis of the purified rHdhNrf2 protein.

Following IPTG induction, the yield of the target protein under cultivation conditions of 20 °C and 37 °C was compared, and the expression levels of the target proteins were analyzed using 12% SDS-PAGE. The results are shown in Figure 7a. pET28a-Nrf2 was expressed in the precipitates at both 20 °C and 37 °C, and no target protein was seen in the supernatant.

The recombinant protein was purified using a HisTrap^TM^ FF Crude nickel column. Gradient elution was performed using 20, 50, and 500 mmol/L imidazole solution, respectively. Crude protein, wash effluent, and elution effluent were treated separately, and samples were collected and subjected to SDS-PAGE (Figure 7b). The results showed that the fusion expression product of the target protein could specifically bind to the nickel ion chelate affinity chromatography column. To further determine the expression of the target proteins, the purified proteins were verified using Western blot, and the hybridization bands were as expected (Figure 7c,d).

## 3. Discussion

In this study, full-length *Nrf2* cDNA was obtained from *H. discus hannai*; the first study to investigate Nrf2/ARE in Gastropoda. A typical bZIP_CNC domain was identified at 570–673 bp in the HdhNrf2 protein. In the *C. gigas* Nrf2 protein, two identical bZIP_CNC domains have been detected [20]. Comparing the predicted HdhNrf2 amino acid sequence with the known *Nrf2* genes of other species, HdhNrf2 showed a higher degree of identity and query coverage with *Cristaria plicata*, which is closely associated with the evolution of the species. Notably, *Drosophila melanogaster* and *Helicoverpa armigera armigera* Nrf2 showed a distant evolutionary relationship with mollusks in the phylogenetic tree, belonging to a third branch of arthropods. This result is similar to that of the phylogenetic analysis of *C. gigas.* Danielli et al. [19].

Nrf2 is an important transcription factor that regulates cellular antioxidant responses and belongs to the CNC transcription factor family [7]. The Nrf2 protein comprises seven conserved Neh (Nrf2-ECH homology) domains: Neh1 to Neh7. Neh1 has a leucine-zipper structure that forms a heterodimer with a small Maf protein. Neh1 is important because the Nrf2 protein needs to recognize and bind to the ARE and thus initiate the transcription of the target gene [23]. The Neh2 region is a binding site for Nrf2 and Keap1. The carboxy-terminal of Neh3 is involved in transcriptional activation by interacting with the transcription coactivator chromo-ATPase/helicase DNA-binding protein (CHD)6 [24]. The Neh4 and Neh5 regions act as transactivation domains that bind to the cAMP response element-binding, protein-binding protein and/or receptor-associated coactivator 3 [25]. Neh6 is a serine-rich protein that regulates Nrf2 stability [26]. The Neh7 is a newly discovered domain which interacts with retinoid X receptor to mediate the repression of Nrf2 [27].

*Nrf2* genes of human and mouse are broadly expressed in various tissues [26]. There are two *Nrf2* genes in zebrafish (*Nrf2a* and *Nrf2b*), each displaying a distinct expression pattern. The expression of *Nrf2a* is highest in the gill and weakly expressed in the ovary, whereas that of *Nrf2b* is most highly expressed in the ovary [14]. Wang et al. [21] detected RpNrf2 expression in *Ruditapes philippinarum* in the gill, mantle, digestive gland, and adductor, and found that the highest transcription level was in the gill and digestive gland. In this study, we investigated HdhNrf2 expression in different tissues. *HdhNrf2* mRNA was expressed in a wide range of tissues and was highest in the hemocytes and gills, followed by the mantle, epipodium, and digestive gland. Mollusks have an innate, non-adaptive system of defense, including cellular and humoral components [28]. Hemocytes are important immune factors, and the gills act as barriers to various exogenous stimuli. The high expression level of *HdhNrf2* mRNA in hemocytes and gills indicates its important effects on immune and antioxidant defenses.

Excessive ROS, including free radicals, such as the superoxide anion (O_2_^−^) and hydroxyl radical (OH), singlet oxygen (1O_2_), and hydrogen peroxide (H_2_O_2_) can cause cellular oxidative damage [2]. To avoid damage to the organism, host cells initiate protective mechanisms by generating antioxidants. The mRNA expression levels of *HdhNrf2* and antioxidant-related genes were determined in H_2_O_2-_treated cells in this study. The transcript levels of *HdhCu/Zn-SOD*, *HdhTPX1*, and *HdhTPX2* were significantly increased (*p* < 0.05) by H_2_O_2_ treatment, suggesting a protective role against oxidative stress initiated by antioxidase production after exposure to H_2_O_2_. A study on *Meretrix meretrix* showed that the Cu/Zn-SOD and Mn-*SOD* mRNA levels were substantially higher in response to H_2_O_2_ challenge in experimental clams than in the controls [29]. TPX and GPX are important antioxidant enzymes that are involved in H_2_O_2_ detoxification. In a previous study, the *AbTPx1* and *AbTPx2* mRNA of *H. discus discus* were upregulated by H_2_O_2_ in the gill and digestive tract tissues, suggesting that both genes are inducible [30]. However, a study on penaeid shrimp revealed that an H_2_O_2_ injection did not induce any upregulation of Prx transcription [31]. De Zoysa et al. [32] reported that mitochondrial thioredoxin-2 from disk abalone was upregulated in the gill and digestive tract tissues by H_2_O_2_ at 3 and 6 h post-oxidative stress in vivo. Kim et al. [33] found that Nrf2 regulates the hemin-induced activation of the thioredoxin gene in K562 cells through ARE. Notably, HdhNrf2 showed no significant response to H_2_O_2_, which may be related to the post-transcriptional mechanisms of Nrf2 [34].

Nrf2 is a key transcription factor that protects the cells from harmful exogenous substances and oxidative damage. The inactivation or deletion of Nrf2 increases the sensitivity of cells to stress, leading to prolonged inflammatory processes, apoptosis, and chemical carcinogenesis, among other pathological processes [35,36,37]. Wang et al. [21] reported the effects of benzo-a-pyrene exposure on Nrf2, Keap1, E3, GST-pi, SOD, CAT, and GPx genes in *Ruditapes philippinarum* and constructed a dsRNA targeting the *Nrf2* gene. The authors observed that 24 h after injecting dsRNA in vivo, the expression level of Nrf2 decreased by 63.2%. Similarly, the expression of downstream regulatory genes was inhibited, suggesting that Nrf2 is vital for the regulation of antioxidant genes [38,39,40].

Nrf2 is a transcription factor that regulates the expression of antioxidant proteins that protect against oxidative damage triggered by injury and inflammation. The acquisition of recombinant protein is crucial. In this study, by constructing the recombinant expression vector and optimizing the expression conditions, we successfully obtained the rHdhNrf2 protein. The molecular weight obtained from the Western blot hybridization is around 100 kDa, which is larger than the predicted theoretical protein molecular weight of 77 kDa. This discrepancy may be related to the post-translational modifications that the protein undergoes, such as glycosylation. However, the role of rHdhNrf2 protein in cellular antioxidant stress and defense mechanisms against inflammation is still under investigation, which is vital for studying its biological functions and potential therapeutic applications.

## 4. Materials and Methods

### 4.1. Experimental Animals

Pacific abalones (*H. discus hannai*) were purchased from the Dongshan abalone farm in Fujian Province and acclimatized in a laboratory environment for 7 d before the experiments were carried out. Animals were reared in 80 L PVC tanks containing 40 L of natural seawater, which was purified through a bio-filter and renewed daily to ensure water quality. Animals were fed the marine alga, *Gracilaria tenuistipitata*, during the acclimation and experimental periods.

### 4.2. H_2_O_2_ Challenge and Preparation of Different Tissues

The abalones were divided into the saline and H_2_O_2_ groups. Abalones were injected with 50 µL of the H_2_O_2_ or an equal volume of sterile saline via the front of the foot. The injection method was based on a previously published studies [41]. Normal Pacific abalone were used as controls. Abalone hemocytes, gills, gonads, mantle, digestive gland, shell muscle, epipodium, and other tissue samples were collected at 0, 3, 6, 12, 24, 48, and 96 h after injection. Samples from five abalones were collected from each group as parallel samples. The samples were rapidly placed in liquid nitrogen and stored at −80 °C.

### 4.3. Cloning of the HdhNrf2 cDNA Sequence

The total RNA was extracted according to the manufacturer’s instructions using the RNAprep pure Tissue Kit (DP431, Tiangen, Beijing, China)., as previously described [42,43]. A partial HdhNrf2 sequence was obtained from the transcriptome library (GenBank accession No. MK848864). The first strand of hemocyte cDNA was synthesized using M-MuLV Reverse Transcriptase (Sangon Biotech, Shanghai, China). The 5′ and 3′ end sequences were obtained using the RACE PCR with 5′/3′ RACE system 2.0 (Invitrogen Life Technologies Inc., Carlsbad, CA, USA) following the manufacturer’s instructions. Nested PCR was carried out with gene-specific primers *HdhNrf2-F1* (outer) and *HdhNrf2-F2* (inner) for 3′ RACE, and *HdhNrf2-R1* (outer) and *HdhNrf2-R2* (inner) for 5′ RACE. The primer sequences used are listed in Table 2 and were obtained from Sangon Biotech. PCR was conducted as follows: 95 °C for 3 min, followed by 33 cycles of 94 °C for 30 s; 58 °C for 30 s; 72 °C for 60 s, and 72 °C for 7 min. The PCR products were cloned into the pMD-18T vector (TaKaRa Bio., Inc., Shiga, Japan) and sequenced (Sangon Biotech, Shanghai, China).

### 4.4. Sequence Analysis

The open reading frame (ORF) was predicted using the DNASIS MAX software (Hitachi DNASIS ^®^ MAX v3.0). Characteristic HdhNrf2 protein signatures were predicted using the ExPASy-Prosite (http://prosite.expasy.org, accessed on 17 November 2024) and the SMART online server (http://smart.embl-heidelberg.de, accessed on 17 November 2024). The ExPASy ProtParam tool (http://web.expasy.org/protparam, accessed on 17 November 2024) was used to predict the mass and p*I* of the HdhNrf2 protein. Conserved domains were analyzed using the NCBI Conserved Domain Search (https://www.ncbi.nlm.nih.gov/Structure/cdd/wrpsb.cgi, accessed on 17 November 2024). A homology search was performed using BLASTN2 and BLASTP on the NCBI Net WWW Server (https://blast.ncbi.nlm.nih.gov/Blast.cgi, accessed on 17 November 2024). Multiple sequence alignments were performed using Clustal W2.0. Phylogenetic relationships between Nrf2 proteins were analyzed using the neighbor-joining method and MEGA7.0 software. The reliability of branching was tested using bootstrap resampling (1000 pseudo-replicates).

### 4.5. Quantitative Real-Time PCR (qPCR)

The total RNA was extracted according to the manufacturer’s instructions using the RNAprep pure Tissue Kit (DP431, Tiangen, Beijing, China), as previously described [42,43]. During the RNA extraction process, DNase I was used to remove genomic DNA, ensuring the purity of the RNA samples. After determining the total RNA concentration using an Infinite200Pro microplate reader (TECAN, Männedorf, Switzerland), 4 µg total RNA was extracted from each tissue, and cDNA was synthesized by reverse transcription using Evo M-MLV Reverse Transcription Reagent Premix (for qPCR; Accurate Biotechnology, China) according to the manufacturer’s instructions. A reaction system without reverse transcriptase is set up to check for the presence of DNA contamination in the RNA samples.

The qPCR system had a volume of 20 µL and included 10 ng of total RNA, 10 pmol of specific primers (Table 1), and 10 µL of FastStart Universal SYBR Green Master (Rox; Roche). The reaction conditions were as follows: preincubation at 95 °C for 600 s, followed by 45 cycles (denaturation, 95 °C for 10 s; annealing, 60 °C for 10 s; and extension at 72 °C for 10 s). Ribosomal proteins L3 and L7 were used as internal reference genes, according to Lee et al. [44,45]. qPCR data were calculated using the 2^−∆∆CT^ method. Melt-curve analysis was performed to analyze the specificity of the PCR products.

### 4.6. Recombinant Expression

The synthetic HdhPGRP-SC2-like gene was cloned into the pET-28a(+) expression vector and transformed into *Escherichia coli* BL21 (DE3) pLysS for recombinant plasmid expression. The pET28a-Nrf2 positive monoclonal strain was incubated at 37 °C and 180 rpm; and when the Optical Density (OD) value reached 0.6, the induction agent, IPTG, was added, and the incubation was continued at 20 °C for 16 h and 37 °C for 4 h. Organisms were collected and centrifuged after full solubilization by sonication, and the supernatant and precipitate were subjected to SDS-PAGE. PAGE electrophoresis was performed on the supernatant and precipitate.

The HdhNrf2 precipitate was collected by the ultrasonic crushing of the bacteriophage and separated and purified using a nickel-ion metal-chelate affinity chromatography column after denaturation. The HdhNrf2 bacteriophage was collected by ultrasonication, and the protein supernatant was directly passed through an affinity chromatography column. The chelated fusion protein was eluted with imidazole, the UV absorption curve was detected at 280 nm, and a clear peak of the eluted protein was observed. The concentration of recombinant protein was determined using the BCA method.

### 4.7. Western Blotting

The methods reported in our previous study were used for Western blotting [45]. The crude recombinant protein was separated by 12% SDS-PAGE and transferred onto a PVDF membrane. It was then blocked with PBST containing 5% skim milk at 37 °C for 2 h, followed by incubation with the mouse anti-His tag monoclonal antibody (1:5000 dilution) at 37 °C for 1 h. After washing with PBST four times, it was incubated with horseradish peroxidase (HRP)-conjugated goat anti-mouse secondary antibody (1:8000 dilution) at 37 °C for 60 min. After washing with PBST four times, the membrane was developed with 3,3′,5,5′-Tetramethylbenzidine (TMB) substrate, and then photographed.

### 4.8. Determination of Antioxidant Enzyme Activity in H. discus hannai

Serum samples were prepared for assessing enzyme activities. For the quantification of various biochemical parameters, we employed commercial assay kits from Nanjing Jiancheng Bioengineering Institute (Nanjing, China), strictly adhering to the manufacturers’ protocols. The levels of malondialdehyde (MDA) were measured employing the thiobarbituric acid (TBA) assay (A003-1). The total antioxidant capacity (T-AOC) was assessed using the ABTS method (A015-2-1). For the activity of superoxide dismutase (SOD), the nitro blue tetrazolium (NBT) method (A001-1) was applied, and catalase (CAT) activity was determined by the ammonium molybdate method (A007-1-1). Comprehensive procedural details can be found within the guidelines that accompany each kit.

### 4.9. Statistical Analysis

The statistical analyses were conducted using SPSS 24.0 (SPSS, Chicago, IL, USA) software. The numbers (n) of samples or replicates are indicated in figure legends and in the Section 4. For bar charts, all values are presented as mean  ±  s.d. Differences in gene expression levels among various tissues were analyzed using multiple-group comparisons. Significance analysis was calculated by one-way ANOVA followed by Tukey’s HSD post hoc test and represented by different letters. For the gene expression differences between the experimental and control groups at each time point, a significance analysis was calculated by two-tailed independent sample *t*-tests, and the F-statistic, degree of freedom, and exact *p* values are presented. The data were initially assessed for adherence to the normal distribution with the Shapiro–Wilk test and equality of variances. A difference of *p* < 0.05 was considered significant (*), a difference of *p* < 0.01 was considered to be very significant (**), and *p* < 0.001 was considered to be extremely significant (***).

## 5. Conclusions

This study investigated the functional characteristics of the Nrf2/ARE signaling pathway in the abalone (*Haliotis discus hannai*) by successfully cloning and analyzing the full-length cDNA sequence of the HdhNrf2 gene. The findings revealed notable differences in the gene’s expression across various abalone tissues, with particularly high levels in the gills, potentially linking to its antioxidant role. Further research, which involved constructing expression vectors and obtaining recombinant proteins, confirmed the crucial function of HdhNrf2 under conditions of oxidative stress. These discoveries offer novel perspectives on the antioxidant mechanisms in abalone and establish a foundation for future studies.

## Figures and Tables

**Table 1:** Sequence information of Nrf2 for multiple sequence alignment and phylogenetic analysis.

Protein	Species	Accession Number	Query Cover	Identity	E-Value
Nrf2	*Cristaria plicata*	AZM32563.1	95%	47%	0.0
Nrf2	*Crassostrea gigas*	EKC19603.1	97%	40%	2 × 10^−112^
Nrf2	*Azumapecten farreri*	AWV55266.1	95%	46.62%	2 × 10^−172^
Nrf2	*Ruditapes philippinarum*	AWV55267.1	71%	44.72%	9 × 10^−126^
Nrf2	*Danio rerio*	NP_878309.1	34%	45.02%	8 × 10^−51^
CNC	*Drosophila melanogaster*	NP_001247256.1	25%	42.50%	1 × 10^−43^
Nrf2	*Mus musculus*	NP_035032.1	67%	42.73%	3 × 10^−46^
Nrf2	*Homo sapiens*	NP_006155.2	55%	52.41%	7 × 10^−47^

**Table 2:** Primer sequence information.

Gene	Usage	Primer Sequences (5′-3′)	GenBank ID
*HdhNrf2-F1*	3′-RACE	TTACAATGTGCATGGATATAACGTTTGCCTG	MK848864
*HdhNrf2-F2*	3′-RACE	TGCCTGGAATGAAGAGACACATTGAAAT	
*HdhNrf2-R1*	5′-RACE	CCCGTATGTGGCATCACGGACTGTA	MK848864
*HdhNrf2-R2*	5′-RACE	GCAACATTTTCGAGGTTTTCAATACCACAA	
*HdhTPX1 QS3*	qPCR	TCAACACTCCACGTGACCAG	MN123623
*HdhTPX1 QA3*	qPCR	GCGTAGGACTCCCTTGTTGT	
*HdhTPX2 QS3*	qPCR	CTGTTGGACGCTCAGTGGAT	MK257743
*HdhTPX2 QA3*	qPCR	AGTAGTTCTGGCTGCCCTTG	
*HdhCu/Zn-SOD QS2*	qPCR	CAGTTCGGGGACAACACCAA	KX302627
*HdhCu/Zn-SOD QA2*	qPCR	TGTTTGCTACTCCTGATGCGT	
*HdhMn-SOD QS3*	qPCR	GGACTGGTTCCCCTCTTTGG	KX302628
*HdhMn-SOD QA3*	qPCR	GCCACATTTTCCCAGTTGGC	
*HdhTrX2 QS2*	qPCR	GGCAAAGCAGGCAAAGTGATT	MN123624
*HdhTrX2 QA2*	qPCR	GCTGACCATTTCTGATGCCC	
*HdhNrf2 QS2*	qPCR	CGAGGCAAACACTACAAGCG	MK848864
*HdhNrf2 QA2*	qPCR	GGGCGACATGCTTTGAGTTG	
*3RPL-FW*	qPCR	TCATTGCACACACCCAGACT	KP698943
*3RPL-RV*	qPCR	CAATGACCTCATCCTGTTCG	
*7RPL-FW*	qPCR	CAAGCTGAACACTCCAAACG	KP698945
*7RPL-RV*	qPCR	TCCACAGCACTGATGTTTCC	

## Data Availability

Data contained within the article.

## Supplementary Materials

The following supporting information can be downloaded at: https://www.mdpi.com/article/10.3390/ijms252212429/s1.
