# Supplementary material for: The Molecular Characterization and Antioxidant Defense of a Novel Nrf2 from the Pacific Abalone Haliotis discus hannai Ino
Source: Int J Mol Sci. 2024 Nov 19;25(22):12429. doi: 10.3390/ijms252212429 (PMC11594797; doi:10.3390/ijms252212429)
Supplement: Supplementary file 1 [file ijms-25-12429-s001.zip › ijms-3288871-supplementary.pdf]

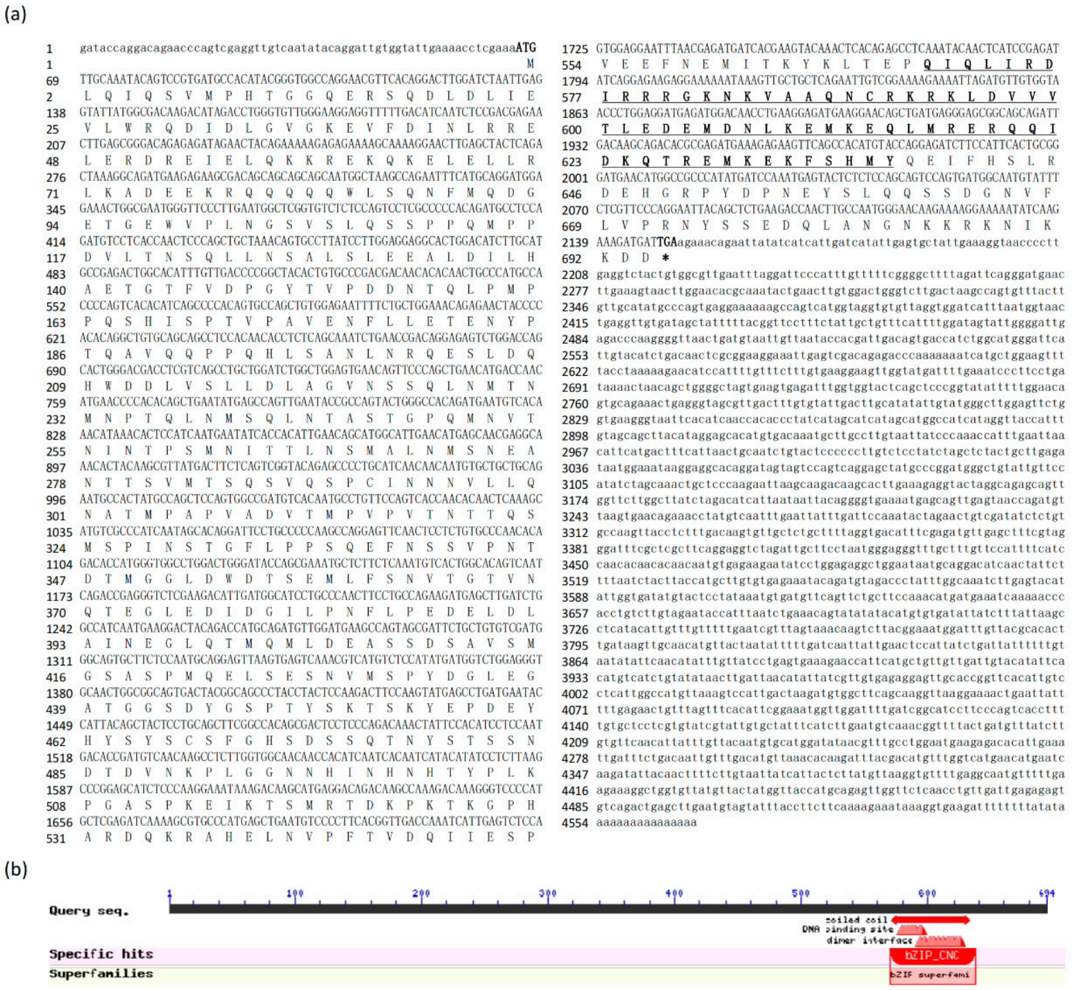

Supplementary Figure S1. Analysis of the *Haliotis discus hannai* Nrf2 protein sequence. (a) Sequence of the full-length cDNA and deduced amino acids of HdhNrf2. The start of the ORF (ATG) is shown in bold, and a stop codon (TAA) is shown in bold and marked with an "*". The numbers on the left of the sequence give the position of the first nucleotide and amino acid on each line. The basic leucine zipper (bZIP) domain of Cap'n'Collar (CNC) transcription factor is underlined in bold. (b) The bZIP domain of Cap'n'Collar transcription factor domain (BZIP_C) of *Haliotis discus hannai* Nrf2 protein.

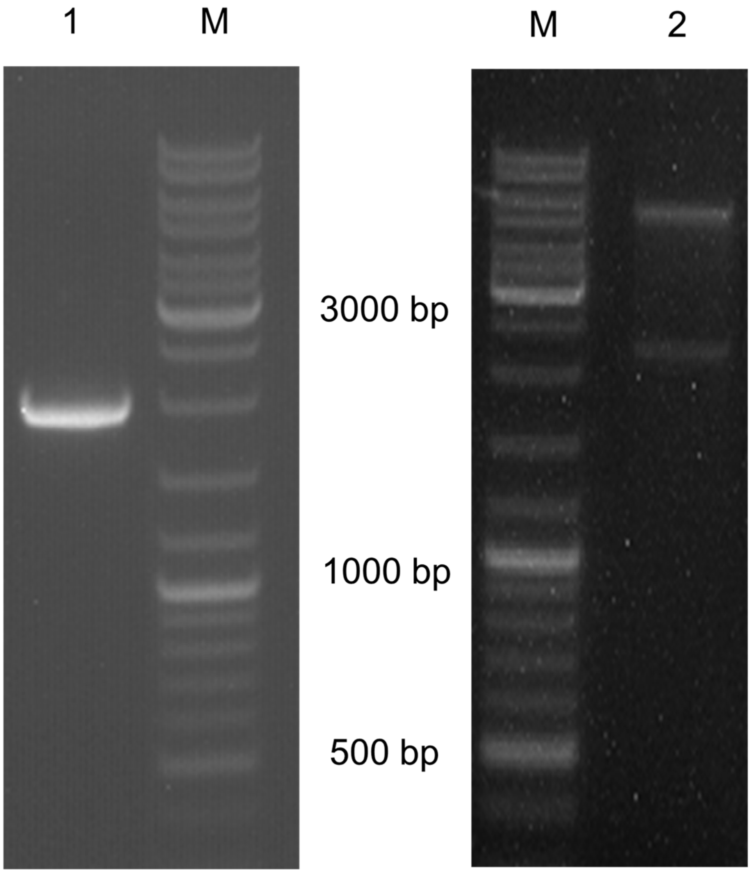

Supplementary Figure S2. Recombinant expression vector construction diagram. Left: Amplification of the target fragment. Right: Enzymatically digested recombinant plasmid. Marker: SM0331, Thermo; 1: Amplification of HdhNrf2 target fragment; 2: Enzymatically digested pET28a-Nrf2 recombinant plasmid.
